# Biomonitoring of heavy metals using *Contracaecum quadripapillatum* (Nematoda) in comparison to its fish host, *Lates niloticus*, from the Nile River, Egypt

**DOI:** 10.1007/s10661-023-11156-2

**Published:** 2023-04-01

**Authors:** Hasnaa Thabit

**Affiliations:** Department of Zoology and Entomology, Faculty of Science, Assiut University, PO 71526 Assiut, Egypt

**Keywords:** Bioaccumulation, Bioindicators, Fish parasites, Freshwater ecosystem, Metal pollution

## Abstract

Recently, fish parasites have been used as a biomonitoring tool to indicate the health status of ecosystems. Therefore, this research aimed to evaluate the potential capacity of *Contracaecum quadripapillatum* larvae as accumulation indicators for metal pollution and compare metal concentrations in host tissues of non-infected and infected fish: *Lates nilotic*us from the Nile River. Accumulations of Cd, Cu, Fe, Mn, Ni, Pb, and Zn in larval nematodes and tissues of the liver, kidney, and muscles of both infected and non-infected fish were determined. All metal concentrations exhibit a significantly higher increase in larval nematodes than the muscles of infected fish and vice versa except Cd in the kidney. On the other hand, only Cd, Mn, Pb, and Zn concentrations were significantly higher in the parasite than in the host liver. Therefore, bioaccumulation factors were most obvious and effective in the muscles of infected fish than in the liver and kidney. *Contracaecum* larvae accumulate Cd and Pb more than other metals. The infrapopulation size of *C. quadripapillatum* correlated with metal concentrations in different host tissues, especially the kidney, while the correlations between metal levels in the tissues of both parasite and fish organs exhibit different patterns in each organ. The current work revealed that *C. quadripapillatum* larvae represent environmental monitors for metal pollution in the freshwater ecosystem.

## Introduction

In both freshwater and marine habitats, fish are stressed by unfavorable human activities (Leite et al., 2016). A large body of research has shown that some fish parasites have a larger capacity for metal accumulation than their host tissues, making them useful as a biomonitoring tool indicating the ecosystem’s environmental quality (Leite et al., 2016; Mehana et al., 2020). Heavy metals are regarded as the most significant aquatic ecosystem pollutants due to their toxicity and ability to accumulate in aquatic organisms (Hassan et al., 2018). They are persistent toxins and cannot be degraded (Ali et al., 2019). Moreover, they may promote more infection with parasites in aquatic species, mainly fish by decreasing the host’s immunological response (Khan & Thulin, 1991). Valuable knowledge is given by parasites on the chemical status of their environments (Beeby, 2001). Fish helminth parasites are sensitive to metal pollution and act as sentinel organisms for degraded ecosystems (Sures et al., 2017). Although parasites have pathogen effects on their hosts, they can sometimes be of benefit to their hosts. They can minimize metal concentrations in their host tissues (Malek et al., 2007; Radwan et al., 2022). However, certain parasite species can accumulate metals in their bodies thousands of times more than the fish organs (Mehana et al., 2020). Parasites react to anthropogenic pollution and can be employed as either accumulation indicators or effect indicators (Sures, 2004; Uçkun, 2017). Also, fish helminth parasites operate as microfilters, extracting metals from their hosts (Najm & Fakhar, 2015). Heavy metals include essential group (e.g., Cu, Fe, and Zn) that play an important role in the metabolism of fish and toxic group (e.g., Cd, Pb, and Hg) with unknown functions in the biological system (Wakawa et al., 2008). Pollutant bioaccumulation in aquatic environments is a dynamic and complicated process that is influenced by organism physiology and environmental physiochemistry (Kapustka, 2004). The bioaccumulation factor gives proof of the period of exposure because the accumulation occurs more quickly in the parasite (Hassan et al., 2018). As a result, higher metal levels in the host muscle and a lower bioaccumulation factor reflect long-term exposure (Sures & Siddall, 2001). The study of Keke et al. (2020) reported high BAF (>1) of all studied metals indicating that the helminths had a comparatively high ability for accumulating metals from host muscles. Depending on the level of metal bioaccumulation, fish trematodes accumulate some heavy metals at lower rates than acanthocephalans and cestodes (Hassan et al., 2018; Najm & Fakhar, 2015). Many research studies have shown that acanthocephalans and cestodes of fish have the greatest ability to accumulate toxic elements in their tissues in comparison to the host tissues (Hassanine et al., 2017; Mehana et al., 2020; Shahat et al., 2011). Also, Nachev et al. (2013) presented that acanthocephalans had the highest accumulation rate for non-essential elements. The bioconcentration factors of Cd and Pb in acanthocephalans seemed to be higher than those in nematodes and trematodes (Al-Hasawi, 2019). In contrast, the parasitic nematodes have a limited capacity to accumulate heavy metals in their tissues, with variable ability from species to species (Khaleghzadeh-Ahangar et al., 2011; Mehana et al., 2020; Nachev et al., 2013; Sures, 2004). So, some researchers regard them as good sentinels in aquatic environments for heavy metal contamination, while others believe that they are unsuitable sentinels for toxic metal pollution in these habitats (Hassan et al., 2016; Nachev et al., 2013; Najm & Fakhar, 2015). Mazhar et al. (2014) reported divergence between the bioaccumulation capacities of two nematodes and those of their host tissues to conclude that *Hysterothalycium reliquens* showed the highest accumulation capacity for essential metals while *Paraphilometroides nemipteri* had a high tendency to accumulate non-essential ones. The rates of metal accumulation in fish nematode larvae have received little research due to the variation in their larval stages and frequency of encapsulation in host bodies, which may affect the absorption of nutrients and pollutants. Leite et al. (2016) reported that *Contracaecum* sp. larvae had good accumulation capacity for both essential and toxic elements and considered it an appropriate model for monitoring contaminated environments by metals. According to the bioconcentration factors, larval nematodes of the genus *Eustrongylides* had the highest accumulation rates for the essential elements (Nachev et al., 2013), while *Contracaecum multipapillatum* appeared to accumulate more Pb than host muscles, *Oreochromis leucostictus* (Otachi et al., 2014). In contrast, *Contracaecum multipapillatum* were inefficient accumulation indicator (Sures, 2003). The present research aims to evaluate the concentrations of some essential (Cu, Fe, Mn, Ni, and Zn) and non-essential metals (Cd and Pb) in *C. quadripapillatum* larvae and tissues of their host *L. niloticus* from Nile River, Assiut governorate, Egypt. Also, to determine metal bioaccumulation in a parasite according to its host, the bioaccumulation factor (BAF) is calculated. Moreover, the present research seeks the correlations between the metal concentrations in the parasitic larvae and tissues of infected fish, and to determine whether the metal concentrations in the parasitic larvae and tissues of infected fish depend on the parasitic infrapopulation size.

## Materials and methods

### Sample collection

**Fig. 1:**
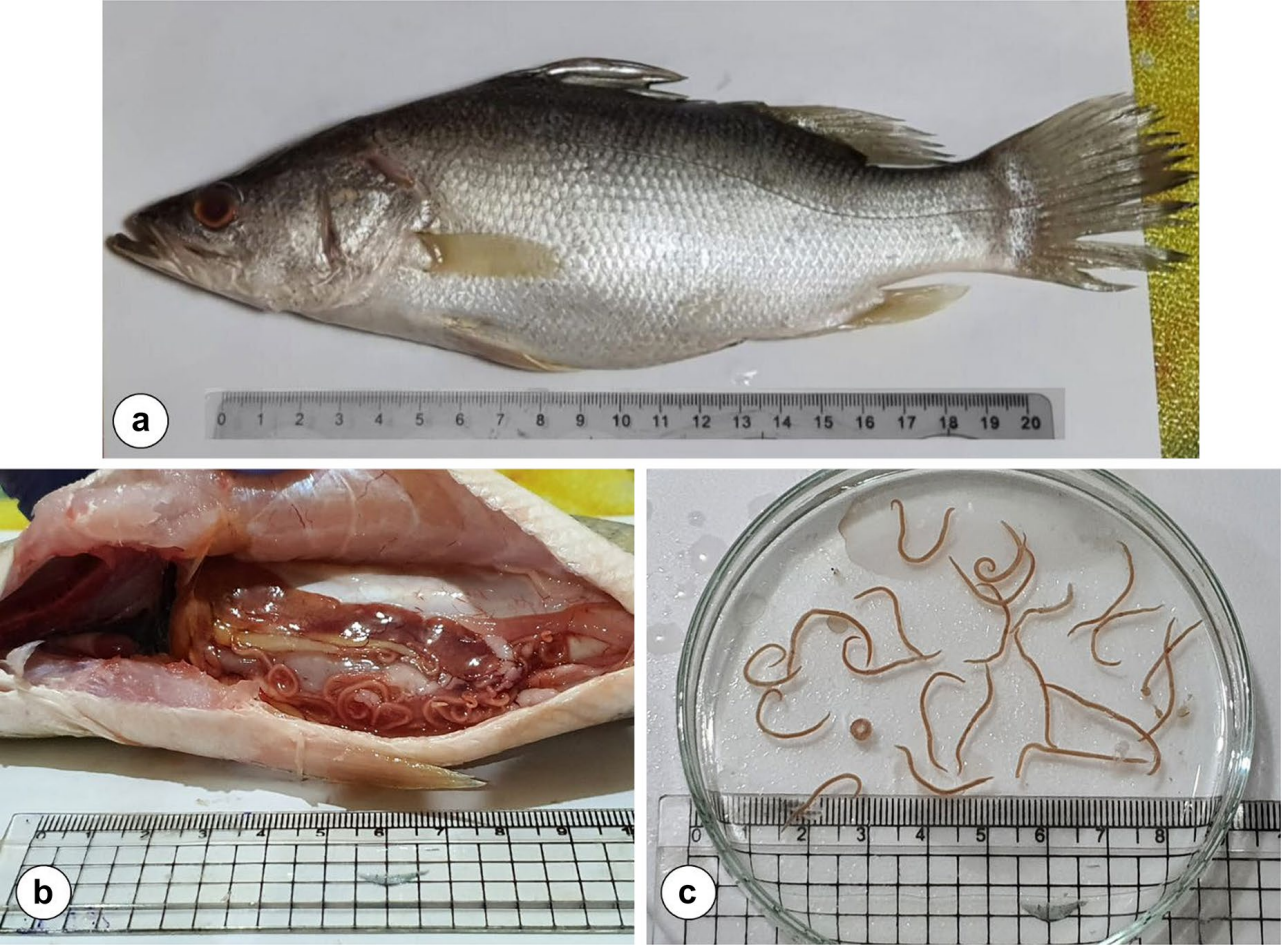
**a** *Lates niloticus* fish, **b** *Lates niloticus* fish infected with *Contracaecum quadripapillatum* larvae, **c** isolated *Contracaecum quadripapillatum* larvae

During the period from September 2020 to March 2021, 112 specimens of *L. niloticus* Linnaeus, 1762 from Assiut Governorate (latitude 27°10′51.46″N, longitude 31°11′1.25″E) on the Nile River, Egypt were collected (Fig. 1a). The selected fish were classified into two groups: non-infected and infected (five samples in each). The non-infected samples weighed between 248 and 380 g (average 311 ± 23.47) g and their standard lengths ranged between 24 and 28 cm (average 25.8 ± 0.75) cm, while the infected samples weighed between 355 and 416 g (average 388.8 ± 11.06) g and their standard lengths ranged between 26 and 28 cm (average 27.1 ± 0.33) cm. Fish were necropsied and scanned for the presence of helminth parasites using a dissecting microscope (Fig. 1b). Nematodes were collected from the abdominal cavity and mesenteries in Petri dishes (Fig. 1c). Thereafter, the worms were identified in a previous study by Thabit and Abdallah (2022). The prevalence of infection was 66.96%. Parasitic infrapopulation size was determined (25–95 larvae/fish). Fish with concurrent infections were ignored in this study to avoid confusion. Samples of dorsal muscles, liver, and kidney of infected and non-infected fish, in addition to the larval nematode, were kept in the freezer at − 20 °C until later processing for trace metal analysis.

### Analysis of heavy metals

Tissue samples from fish and parasites were analyzed using Zimmermann et al. (2001)’s technique. The frozen tissues were first left to defrost, then they were sliced into little pieces and dried for 24 h at 70 °C. Then they weighed (0.5 g liver and muscles, 0.05 g kidney, 0.14 g parasite) and digested in 20 ml of a mixture of HNO3 (65%) and HCLO4 (3:1). Specimens were heated to speed digestion. The aqueous layer was then diluted with deionized water to 100 ml. The heavy metal concentrations (Cd, Cu, Fe, Mn, Ni, Pb, and Zn) were measured using inductively coupled plasma-optical emission spectrometry (ICP-OES) (ICAP 6200) at the Central Laboratory, Faculty of Agriculture, Assiut University. The metal concentrations were represented as micrograms per gram (dry weight) in parasite and fish tissues.

### Data analysis

The data analyses were done using SPSS software (version 25). Data were displayed as mean ± standard error. The BAFs and the ratio of heavy metal concentration in the parasite to that in host organs were calculated using the following equation according to Sures et al. (1999).

$$\mathrm{BAF}=\mathrm C\;\left[\mathrm{parasite}\right]\;/\;\mathrm C\;\lbrack\mathrm{host}\;\mathrm{tissue}\rbrack$$

The significant variations in heavy metal values between infected and non-infected fish were examined using an independent-samples *t*-test (two-tailed). In the case of normally distributed data, one-way ANOVA was used to look for significant differences between heavy metal concentrations in parasitic larvae and infected fish tissues followed by the Duncan test to discover different deviations between means. For non-normal data, the Kruskal–Wallis test was used. When there were significant differences, a series of the Mann–Whitney *U* test was done to show the differences. Pearson correlation was used to investigate a possible association between metal levels in the parasitic nematode and different tissues of infected fish, as well as the association between the parasite number and metal levels in the parasitic nematode, and in the selected tissues of infected fish.

## Results

The investigated metals and their distribution in the examined fish are presented in Table 1. The results demonstrated that the highest metal concentrations were found in the kidney followed by the liver and then the muscles in both infected and non-infected groups except Fe in non-infected fish whose highest concentration was detected in the liver, immediately followed by the kidney and then the muscles. The liver of infected fish contained significantly lower concentrations of Cd, Cu, Fe, Pb, and Zn. Although mean Mn and Ni concentrations in an infected fish were higher than those in non-infected fish, their concentrations were not significant. Comparisons of metal concentrations in the kidney of non-infected and infected fish showed different patterns; in the infected host, Cd and Cu concentrations were significantly lower than in non-infected fish. Otherwise, certain metals including Ni, Zn, and Fe were found at significantly higher levels in infected fish. In addition, non-significant differences in Mn and Pb concentrations were detected between infected and non-infected fish, despite their higher mean concentrations in infected fish. In the muscles, the levels of Fe and Pb were detected in lower concentrations in infected samples while Zn was found in higher concentrations. These values were significant. The remaining metal concentrations in non-infected fish were higher than in infected ones, but these differences were not significant. It is noticeable that the Mn concentrations in the liver, kidney, and muscles have non-significant values between non-infected and infected fish.

**Table 1:** Mean metal concentrations (µg/g dry weight) ± standard error in selected tissues of infected and non-infected fish, *Lates niloticus*

	**Liver**				**Kidney**				**Muscles**			
	**Non-infected**	**Infected**	***T*-test**	***P* value**	**Non-infected**	**Infected**	***T*-test**	***P* value**	**Non-infected**	**Infected**	***T*-test**	***P* value**
**Cd**	0.26 ± 0.04	0.09 ± 0.02	3.418	0.002	2.24 ± 0.50	0.67 ± 0.11	3.069	0.005	0.13 ± 0.04	0.07 ± 0.01	1.57	0.128
**Cu**	33.34 ± 4.11	15.41 ± 0.75	4.291	< 0.0001	70.17 ± 14.70	30.56 ± 1.57	2.68	0.012	4.64 ± 1	2.64 ± 0.19	1.98	0.058
**Fe**	791.60 ± 36.62	431.51 ± 32.87	7.317	< 0.0001	766.09 ± 79.15	8385.52 ± 2980.39	2.556	0.016	728.36 ± 157.21	200.31 ± 32.24	3.29	0.003
**Mn**	5.87 ± 0.19	6.20 ± 0.15	1.316	0.199	39.53 ± 4.53	45.23 ± 4.19	0.922	0.364	4.92 ± 0.72	4.28 ± 0.17	0.867	0.393
**Ni**	6.89 ± 0.21	16.84 ± 5.51	1.803	0.082	37.22 ± 12.27	80.95 ± 9.50	2.818	0.009	6.73 ± 0.97	5.93 ± 0.26	0.801	0.43
**Pb**	2.68 ± 0.21	1.88 ± 0.25	2.473	0.020	12.09 ± 2.35	13.25 ± 1.17	0.441	0.663	1.69 ± 0.44	0.65 ± 0.10	2.31	0.028
**Zn**	103.57 ± 8.20	61.53 ± 2.95	4.825	< 0.0001	286.97 ± 30.84	488.14 ± 62.83	2.874	0.008	11.87 ± 0.69	15.99 ± 0.85	3.775	0.001

**Fig. 2:**
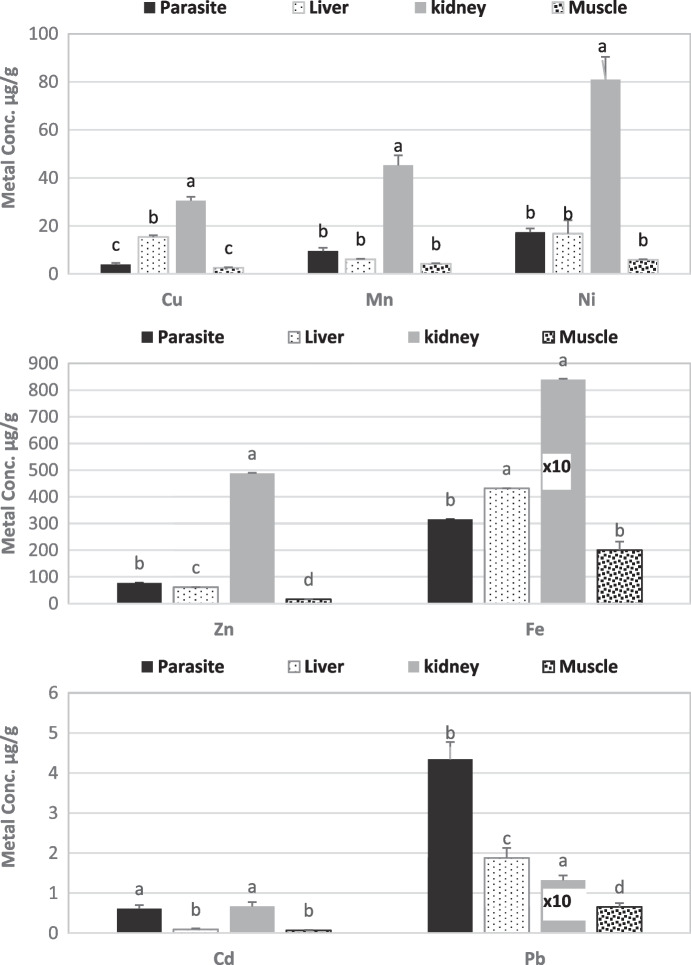
Mean metal concentrations (µg/g dry weight ± standard error) in tissues of *Lates niloticus* and its parasite *Contracaecum quadripapillatum*. (× 10 = the value multiplied by 10, similar characters refer to no significant differences)

*Contracaecum* larvae had metal concentrations in the following order: Fe ˃ Zn ˃ Ni ˃ Mn ˃ Pb ˃ Cu ˃ Cd, whereas the liver had the following order: Fe ˃ Zn ˃ Ni ˃ Cu ˃ Mn ˃ Pb ˃ Cd; in both kidney and muscles was Fe ˃ Zn ˃ Ni ˃ Mn ˃ Cu ˃ Pb ˃ Cd (Fig. 2). The concentrations of Cd, Mn, Pb, and Zn were found to be significantly higher in larvae of *C. quadripapillatum* when compared with the host liver, while the liver has the highest levels of Fe and Cu. No significant differences were detected in Ni levels among the parasite and liver. *Contracaecum* larvae presented significantly lower concentrations in all analyzed metals than kidney tissue except Cd which had non-significant results. On the contrary, in *Contracaecum* larvae, mean concentrations of all analyzed metals were reported at significantly higher levels than those in the muscles (Fig. 2).

The BAFs showed the ability of the parasite to accumulate metals relative to other host tissues under study as shown in Table 2. Except for Cu and Fe, all metals had higher mean BAFs in *Contracaecum* larvae (˃1) than in the host liver. The kidney BAFs had low levels for all metals (< 1) except for Cd whose concentration in the parasite was 1.4-fold higher than in the kidney. The bioaccumulation factors for all metals were most obvious and effective in the muscles of infected fish than in the liver and kidney. In comparison to the parasite, the muscles had the significantly lowest levels of all heavy metals. Therefore, the BAF values were higher in the muscles than those in the liver and kidney. According to the BAFs, the *Contracaecum* larvae accumulated Cd and Pb at the highest rates, followed by Zn, Ni, Mn, Fe, and Cu, since the parasite’s levels of Pb and Cd were more than 12-fold compared to those in the host muscles. The mean BAFs for Zn, Fe, Mn, and Ni showed significant differences between the liver, kidney, and muscles of *L. niloticus*, while the mean BAFs for both Cu and Pb were significantly higher in the muscles than in the liver and kidney. For Cd, non-significant results were reported between mean BAFs in the liver and muscles but there were significant results between them and in the kidney.

**Table 2:** Mean bioaccumulation factors (*BAF* = *C*[*parasite*] / *C*[*host tissue*]) ± standard error calculated for parasite *Contracaecum quadripapillatum* for host tissues of *Lates niloticus*

Heavy metals	BAF			
	Liver	Kidney	Muscles	*F* value
Cd	10.11 ± 2.27^b^	1.42 ± 0.36^a^	12.23 ± 2.60^b^	8.17**
Cu	0.27 ± 0.05^a^	0.14 ± 0.02^a^	1.64 ± 0.25^b^	32.301**
Fe	0.82 ± 0.11^b^	0.09 ± 0.01^a^	1.82 ± 0.23^c^	35.121**
Mn	1.54 ± 0.20^b^	0.23 ± 0.04^a^	2.20 ± 0.25^c^	28.367**
Ni	2.12 ± 0.35^b^	0.23 ± 0.02^a^	3.02 ± 0.30^c^	28.439**
Pb	2.82 ± 0.44^a^	0.34 ± 0.03^a^	12.21 ± 3.50^b^	9.435**
Zn	1.29 ± 0.10^b^	0.19 ± 0.02^a^	5.05 ± 0.41^c^	110.507**

One-way ANOVA followed by the Duncan test

^**^Significant at the 0.01 level (similar superscript letters refer to no significant differences)

Table 3 shows the Pearson correlation coefficients between metal concentrations in the parasite and fish tissues. Correlation analyses revealed that there were no significant relationships between the metal concentrations of the parasite tissue and corresponding those of fish liver. However, significant positive correlations were found between Ni concentration in parasite tissue and both Mn and Zn concentrations in fish liver and between Mn concentration in parasite tissue and Zn concentration in fish liver, while a significant negative correlation was found between Ni concentration in the parasite tissue and Cu concentration in the fish liver (Table 3). In the kidney, there were significant positive correlations between Ni, Pb, and Zn concentrations in the parasite tissue and corresponding those in the kidney of infected fish. In addition, significant positive and negative correlations were obtained between different metal concentrations in the parasite tissue and kidney of infected fish (Table 3). In the muscles, there was a significant positive correlation between Mn concentration in the parasite tissue and Mn concentration in fish muscles, while a significant negative correlation was reported between Pb concentration in parasite tissue and Pb concentration in the fish muscles.

**Table 3:** Pearson correlation coefficients (*r*) between metal concentrations in tissues of the parasite and its host *Lates niloticus*

**Fish organ**		**Parasite**													
		**Cd**		**Cu**		**Fe**		**Mn**		**Ni**		**Pb**		**Zn**	
		*r*	*P* value	*r*	*P* value	*r*	*P* value	*r*	*P* value	*r*	*P* value	*r*	*P* value	*r*	*P* value
**Liver**	**Cd**	0.392	0.148	− 0.442	0.099	− 0.257	0.355	0.382	0.160	− 0.182	0.517	0.484	0.068	− 0.182	0.516
	**Cu**	0.380	0.163	− 0.209	0.454	− 0.064	0.821	− 0.500	0.058	** − 0.593**	**0.020**	− 0.042	0.882	− 0.306	0.267
	**Fe**	0.396	0.144	0.058	0.836	− 0.090	0.751	0.230	0.409	0.242	0.385	0.292	0.292	− 0.125	0.658
	**Mn**	0.070	0.804	0.182	0.516	0.313	0.256	0.357	0.192	**0.763**	**0.001**	0.094	0.739	− 0.037	0.895
	**Ni**	0.246	0.376	0.063	0.824	0.131	0.641	0.144	0.610	0.395	0.145	0.170	0.545	− 0.170	0.545
	**Pb**	0.484	0.068	0.011	0.968	− 0.092	0.743	0.103	0.715	0.099	0.725	0.277	0.318	− 0.185	0.509
	**Zn**	0.328	0.233	− 0.026	0.925	0.039	0.889	**0.525**	**0.044**	**0.575**	**0.025**	0.413	0.126	− 0.139	0.620
**Kidney**	**Cd**	− 0.368	0.177	− 0.420	0.119	** − 0.627**	**0.012**	** − 0.583**	**0.023**	− 0.464	0.082	** − 0.539**	**0.038**	− 0.257	0.354
	**Cu**	0.235	0.400	0.101	0.720	0.481	0.069	0.408	0.131	0.396	0.144	− 0.260	0.349	0.167	0.551
	**Fe**	**0.690**	**0.004**	**0.553**	**0.033**	0.510	0.052	**0.701**	**0.004**	**0.668**	**0.007**	**0.747**	**0.001**	**0.860**	** < 0.0001**
	**Mn**	**0.539**	**0.038**	0.475	0.074	0.199	0.478	0.240	0.390	0.358	0.190	**0.883**	** < 0.0001**	0.465	0.081
	**Ni**	**0.713**	**0.003**	**0.609**	**0.016**	**0.540**	**0.038**	**0.634**	**0.011**	**0.677**	**0.006**	**0.853**	** < 0.0001**	**0.756**	**0.001**
	**Pb**	**0.774**	**0.001**	0.487	0.065	**0.574**	**0.025**	**0.723**	**0.002**	**0.698**	**0.004**	**0.644**	**0.010**	**0.824**	** < 0.0001**
	**Zn**	**0.561**	**0.029**	0.395	0.145	0.160	0.569	0.315	0.253	0.414	0.125	**0.806**	** < 0.0001**	**0.686**	**0.005**
**Muscles**	**Cd**	0.164	0.560	0.251	0.367	0.378	0.164	− 0.383	0.158	**0.707**	**0.003**	0.196	0.483	0.434	0.106
	**Cu**	0.242	0.385	0.124	0.659	− 0.051	0.856	− 0.217	0.438	0.105	0.710	0.061	0.829	− 0.219	0.432
	**Fe**	**0.525**	**0.044**	0.069	0.806	0.458	0.086	0.153	0.585	− 0.027	0.925	** − 0.706**	**0.003**	** − 0.573**	**0.025**
	**Mn**	0.230	0.410	0.039	0.890	0.179	0.524	**0.617**	**0.014**	− 0.171	0.543	− 0.397	0.142	− 0.088	0.756
	**Ni**	0.441	0.100	0.129	0.647	0.301	0.276	0.366	0.180	− 0.111	0.692	** − 0.563**	**0.029**	− 0.418	0.121
	**Pb**	0.453	0.090	0.093	0.741	0.407	0.132	0.064	0.822	− 0.008	0.978	** − 0.653**	**0.008**	** − 0.570**	**0.027**
	**Zn**	0.432	0.108	0.162	0.564	**0.527**	**0.044**	0.338	0.219	0.136	0.629	** − 0.577**	**0.024**	− 0.219	0.433

Bold cells indicate significant values of correlation (*r*)

Pearson correlation coefficients between the parasite number of *C. quadripapillatum* and metal concentrations in the parasite and fish tissues are shown in Table 4. The number of parasitic larvae was associated positively with Fe, Ni, and Pb concentrations and negatively with the Cd concentration. However, there were non-significant associations between* C. quadripapillatum* infrapopulation size and fish liver. Moreover, the number of parasitic larvae showed significant positive associations with Cd, Fe, Mn, Ni, and Zn in the kidney and significant negative associations with Pb and Zn concentrations in the muscles.

**Table 4:** Pearson correlation coefficients (*r*) between parasite number of *Contracaecum quadripapillatum* and metal concentrations in tissues of parasitic larva and its host, *Lates niloticus*

Parasite number	Parasite tissue							
		**Cd**	**Cu**	**Fe**	**Mn**	**Ni**	**Pb**	**Zn**
	*r*	** − 0.558**	0.378	**0.727**	0.231	**0.629**	**0.743**	0.343
	*P* value	**0.031**	0.165	**0.002**	0.406	**0.012**	**0.002**	0.210
	**Liver**							
	*r*	0.392	0.043	− 0.344	− 0.233	− 0.420	0.124	− 0.093
	*P* value	0.149	0.878	0.210	0.403	0.119	0.659	0.742
	**Kidney**							
	*r*	**0.537**	0.500	**0.774**	**0.977**	**0.759**	0.308	**0.818**
	*P* value	**0.039**	0.058	**0.001**	** < 0.0001**	**0.001**	0.264	** < 0.0001**
	**Muscles**							
	*r*	0.480	− 0.034	0.297	− 0.299	− 0.129	** − 0.626**	** − 0.882**
	*P* value	0.070	0.905	0.282	0.279	0.647	**0.013**	** < 0.0001**

Bold cells indicate significant values of correlation (*r*)

## Discussion

The current study evaluated the potential accumulation of some essential and non-essential heavy metals in third-stage larvae of *C. quadripapillatum* compared to their host tissues of *L. niloticus*. Previous studies revealed that parasitic larval nematodes, which were extracted from the body cavity, absorb metals at the highest rates (Nachev & Sures, 2015; Sures & Siddall, 1999). Helminth fish parasites aid their hosts’ survival in the presence of heavy metal contamination by accumulating larger amounts of these metals serving as metal sinks as a result (Eissa et al., 2012; Marcogliese et al., 2006; Radwan et al., 2022). Several studies have demonstrated that helminth parasites are an excellent biomarker of heavy metal injuries in the aquatic environment as Khaleghzadeh-Ahangar et al. (2011), Eissa et al. (2012), Tellez and Merchant (2015), Sures et al. (2017), Vidal-Martínez and Wunderlich (2017), and Hassan et al. (2018). Most studies focused on the accumulation potential of adult parasites, while information about the accumulation capacity of larval nematodes is less (Nachev et al., 2013). Pascual and Abollo (2003) noted that larvae accumulate Cd, Cu, Pb, and Zn at higher rates than adults due to the nematode’s larval cuticle’s simpler structure compared to its adult cuticle (Bird & Bird, 2012). According to Szefer et al. (1998) and Mazhar et al. (2014), cuticle morphology appears to have a significant role in absorbing chemicals from the environment.

Fish with helminth infections had lower metal concentrations than non-infected fish (Azmat et al., 2008; Bergey et al., 2002; Sures & Siddall, 1999). The present findings of metal analyses showed that fish tissues (liver, kidney, and muscles) contained non-essential and essential metals like Cd, Cu, Fe, Mn, Ni, Pb, and Zn in both infected and non-infected hosts with non-significant values for Mn. But Cd, Cu, Fe, Pb, and Zn concentrations were significantly lower in the liver of infected fish when compared with that of the non-infected host. Also, some essential and non-essential metals such as Cu and Cd were present at significantly lower levels in the kidney of infected fish, while Fe and Pb concentrations were lowest in the muscles of infected fish. Studies by Brotheridge et al. (1998) and Khaleghzadeh-Ahangar et al. (2011) revealed non-significant results in the concentrations of Pb and Cd between analyzed tissues of infected and non-infected hosts. It is possible that the few parasitic nematodes present within every host, as well as elevated metal levels in the host tissues and environment, were the reasons why no reduction in metal concentration was observed in infected hosts (Khaleghzadeh-Ahangar et al., 2011). Al-Hasawi (2019) found limited Cd and Pb accumulation in fish due to infection with *Procamallanus elatensis* which had a limited ability to accumulate metals showing low bioconcentration factors and is considered a weak bioindicator. In muscles of both present non-infected and infected fish, the heavy metal concentrations were lower than kidney and liver to be as follows: kidney > liver > muscles. Khaleghzadeh-Ahangar et al. (2011) reported lower Cd concentration in the host muscles than in the rest of the tissues examined. Metals accumulated mostly in organs with high metabolic activities, such as the kidney, liver, intestines, and gills (Dural et al., 2006; Kelle et al., 2018; Kenšová et al., 2010), and less so in tissues like muscles that have relatively low metabolic activities (Al-Kahtani, 2009; Canli et al., 1998; Heath, 2018; Roesijadi, 1994).

In the present study, all analyzed metal concentrations were found in *Contracaecum* larvae with significantly higher values than in host muscles. Also, Eira et al. (2008) found Cd, Cu, Ni, Pb, and Zn in higher levels in the nematode, *Anguillicola crassus*, than in the host muscles, *Anguilla anguilla*. Furthermore, Morsy et al. (2012) reported that anisakid juveniles accumulate more Cd, Cu, Fe, Mn, Ni, and Zn except for Pb than the host muscles. Moreover, Bamidele and Kuton (2016) revealed that intestinal nematodes gathered more heavy metals (Cu, Fe, Ni, and Pb) than their host muscles. In contrast, a significantly lower Pb concentration was obtained from *Angullicola crassus* than in the host muscles by Sures et al. (1994) and Palíková and Baruš (2003). The metal concentrations in fish muscles and those of helminth parasites were shown to be non-significantly correlated, according to Keke et al. (2020). The increases in metals in fish muscles following infection may be the result of the host’s compensating response (Samuel & George, 2000). In addition, the levels of Cd, Mn, Pb, and Zn in the current nematode larvae exceeded those in the host liver, while Morsy et al. (2012) found that Cd, Fe, and Mn concentrations were higher in nematodes. The present findings correspond to those obtained by Leite et al. (2016) except that Mn was highest in the host liver; however, in both studies, Fe was highest in the host liver. Some parasites have higher concentrations of heavy metals than their hosts, which is likely a result of the parasites’ failure to synthesize specific organic substances, such as organo-metallic complexes that contain heavy metals (Khaleghzadeh-Ahangar et al., 2011). The nematode larvae absorb essential metals to accomplish rapid development and growth (Hassan et al., 2016; Nachev et al., 2013). Consistent with Eira et al. (2008), the measured metals in the kidney of infected fish were in higher concentrations than larval nematodes. They showed that the Cd, Cu, Pb, and Zn concentrations were higher in the kidney except for Ni which was higher in parasitic nematodes. In addition, Hassan et al. (2013) reported lower metal concentrations in the nematodes *Contracaecum* sp. and *Raphidascaris* sp. compared to fish intestinal walls indicating the limited efficacy of these worms to accumulate metals. This finding might help to explain why there are more metals in diseased fish. In contrast, Mazhar et al. (2014) showed higher metal concentrations in parasitic nematodes than in the kidney of infected fish.

In aquatic ecosystems, when metals bioaccumulation especially non-essential exceeds the permissible standard limits, it will cause a serious threat to the food chain and then to human health (Rauf & Javed, 2007). Therefore, the metal concentrations in muscles (edible part) of both non-infected and infected fish, *L. niloticus*, were compared to the maximum permissible limits advised by the Egyptian Organization for Standardization and Quality (EOSQ), World Health Organization (WHO), and Food and Agriculture Organization (FAO) (Table 5). The concentrations of Cu, Mn, Ni, Pb, and Zn were below the permissible levels of national standards (EOSQ), while Cd and Fe were beyond them. Additionally, Cd, Cu, Ni, and Zn concentrations were under permissible limits of international standards (WHO), while Fe, Mn, and Pb were above them but Pb was below FAO permissible levels. According to Abdel-Mohsien and Mahmoud (2015), consuming Nile *Oreochromis niloticus* is not harmful to humans, even though heavy metal levels in some Egyptian fish samples are higher than the maximum permissible standard limits. Also, previous studies found that trace metals in the muscles of tilapia were within the safe limits and did not pose a risk to public health (Ezzat et al., 2012; Rashed, 2001).

**Table 5:** Maximum permissible limits of heavy metals in fish muscles (mg/kg dry weight) according to the national and international standards

**Standards**	**Cd**	**Cu**	**Fe**	**Mn**	**Ni**	**Pb**	**Zn**	**References**
FAO	-	30	180	-	55	2	-	Hossain et al. (2022)
WHO	1.0	30	109	1.0	30	0.5	-	Hossain et al. (2022), Abah et al. (2016)
FAO/WHO	-	30	333.3	-	-	1.5	100	Hossain et al. (2022), Abah et al. (2016)
EOSQ	0.05	20	30	10	10	2	40	Ibrahim et al. (2016)

The bioaccumulation factors for all metals in the current study were most obvious and effective in the muscles of infected fish than in the kidney and liver. Also, the current parasite showed the highest accumulation rates for Cd and Pb. According to Leite et al. (2016), *Contracaecum* larvae accumulate higher concentrations of toxic elements, which corroborates the present results. Keke et al. (2020) did not record Pb in fish flesh, *Raimas nigeriensis*; however, it was reported in the helminths due to the intestinal parasites easily ingested organo-metallic-bile complexes from their hosts to reduce host metallic accumulation capacity (Al-Hasawi, 2019; Mehana et al., 2020). Sures (2004) mentioned that while some nematodes may not be appropriate as sentinel species, others may serve as bioindicators of environmental pollution. Otachi et al. (2014) indicated lower metal concentrations in *C. multipapillatum* than in the host tissues and therefore was not an efficient accumulation bioindicator. These variations in the ability of nematodes to accumulate metals may be caused by the parasite’s stage of development, where it is located within the host, the length of time a parasite spends in a specific host, how it feeds and excretes, and competition among parasites from the same infra-community or in the host-parasite interaction (Bergey et al., 2002; Nachev et al., 2013; Otachi et al., 2014; Sures, 2003; Sures et al., 1999).

In the present analysis, non-significant associations between the metal concentrations of the parasite and corresponding those of fish liver were found, while there were significant positive associations between some metals such as Ni, Pb, and Zn in parasite tissue and their concentrations in the fish kidney. On the other hand, some metal concentrations in parasite tissue such as Mn and Pb exhibit positive and negative associations respectively with their concentrations in the fish muscle. Al-Hasawi (2019) reported non-significant relationships between Pb and Cd levels in fish intestines and their concentrations in *Procamallanus elatensis* or between its infrapopulation size indicating the absence of interspecific competition (between the parasite and fish) and intraspecific competition (between parasite individuals) for absorbing these metals. Also, the present parasitic larvae record intraspecific competition for accumulating metals whereas there were significant associations between parasite infrapopulation size and Cd, Fe, Ni, and Pb concentrations in parasite tissues. Also, the present findings showed the absence of interspecific competition between metal levels in the liver of infected fish and parasite infrapopulation size. In contrast, the present results recorded a significant negative relationship for Pb and Zn in the muscle and a significant positive relationship for Cd, Fe, Mn, Ni, and Zn in the kidney with *C. quadripapillatum* infrapopulation size. Nachev et al. (2013) found significant positive correlations between Cd, Pb, and Fe concentrations in larval nematodes *Eustrongylides* sp. and the host muscles and liver. Mazhar et al. (2014) reported significant positive correlations between Cd, Pb, and Mn concentrations in fish organs and those in two examined nematodes *Hysterothalycium reliquens* and *Paraphilometroides nemipteri* in addition to Zn in the last nematode. The negative correlation between heavy metals reveals an antagonistic effect on their bioconcentration by fish tissues and their parasites, whereas the positive significance reveals a synergistic effect on the uptake and bioconcentration of those metals inside the studied tissues (AbdAllah, 2006; Otitoloju, 2002). According to Ravera et al. (2007), the metabolic analogy between some metal pairs and/or the seasonal fluctuations may be to blame for the positive correlation. The negative correlation, on the other hand, could be attributed to competitive inhibition of metabolic sites. Furthermore, Shahat et al. (2011) demonstrated that the correlation between metals can be linked to a variety of external and/or internal factors, including physicochemical parameters, and physiological and behavioral conditions concerning the hosts and their parasites.

## Conclusions

In this work, the ability of *C. quadripapillatum* larvae infecting *L. niloticus* to bioaccumulate heavy metals was presented for the first time. To summarize the main findings of this study, the results showed that present larvae were capable of reducing heavy metal concentrations in fish tissues by accumulating them especially given that they tended to accumulate non-essential metals more strongly than essential ones. As a result, the larvae serve as an effective monitoring device for polluted surroundings. So, it further supported the idea that helminthic infections could be used as early-warning signs of heavy metal pollution. Moreover, fish muscles displayed lower metal concentrations than other investigated tissues which is significant from the perspective of food safety given that fish muscles are consumed by humans.

## Data Availability

All data generated by this study are included in the paper and can be obtained from the corresponding author upon approval of a request.

## References

[CR1] Abah J, Mashebe P, Onjefu S (2016). Preliminary assessment of some heavy metals pollution status of Lisikili River Water In Zambezi Region, Namibia. International Journal of Environment and Pollution Research.

[CR2] AbdAllah A (2006). Effects of dissolved lead and copper on the freshwater *prosobranch Lanistes carinatus*. MALACOLOGIA-PHILADELPHIA.

[CR3] Abdel-Mohsien HS, Mahmoud MA (2015). Accumulation of some heavy metals in *Oreochromis niloticus* from the Nile in Egypt: Potential hazards to fish and consumers. Journal of Environmental Protection.

[CR4] Al-Hasawi, Z. M. (2019). Environmental parasitology: Intestinal helminth parasites of the siganid fish *Siganus rivulatus* as bioindicators for trace metal pollution in the Red Sea. *Parasite, 26*. 10.1051/parasite/2019014 PMC6402366 30838973

[CR5] Al-Kahtani MA (2009). Accumulation of heavy metals in tilapia fish (*Oreochromis niloticus*) from Al-Khadoud Spring, Al-Hassa, Saudi Arabia. American Journal of Applied Sciences.

[CR6] Ali, H., Khan, E., & Ilahi, I. (2019). Environmental chemistry and ecotoxicology of hazardous heavy metals: Environmental persistence, toxicity, and bioaccumulation. *Journal of Chemistry, 2019*.

[CR7] Azmat R, Fayyaz S, Kazi N, Mahmood SJ, Uddin F (2008). Natural bioremediation of heavy metals through nematode parasite of fish. Biotechnology.

[CR8] Bamidele A, Kuton MP (2016). Parasitic diseases and heavy metal analysis in *Parachanna obscura* (Gunther 1861) and *Clarias gariepinus* (Burchell 1901) from Epe Lagoon, Lagos, Nigeria. Asian Pacific Journal of Tropical Disease.

[CR9] Beeby A (2001). What do sentinels stand for?. Environmental Pollution.

[CR10] Bergey L, Weis JS, Weis P (2002). Mercury uptake by the estuarine species *Palaemonetes pugio* and *Fundulus heteroclitus* compared with their parasites, *Probopyrus pandalicola* and *Eustrongylides* sp. Marine Pollution Bulletin.

[CR11] Bird AF, Bird J (2012). The structure of nematodes.

[CR12] Brotheridge, R. M., Newton, K. E., & Evans, S. W. (1998). Presence of a parasitic nematode (*Eustrongylidies* sp.) in brown trout (*Salmo trutta*) from a heavy metal contaminated aquatic ecosystem. *Chemosphere, 37*(14–15), 2921–2934. 10.1016/s0045-6535(98)00333-6 9839406

[CR13] Canli M, Ay Ö, Kalay M (1998). Levels of heavy metals (Cd, Pb, Cu, Cr and Ni) in tissue of *Cyprinus carpio*, *Barbus capito* and *Chondrostoma regium* from the Seyhan River, Turkey. Turkish Journal of Zoology.

[CR14] Dural M, Lugal Göksu M, Özak AA, Derici B (2006). Bioaccumulation of some heavy metals in different tissues of *Dicentrarchus labrax* L, 1758, *Sparus aurata* L, 1758 and *Mugil cephalus* L, 1758 from the Camlik lagoon of the eastern cost of Mediterranean (Turkey). Environmental Monitoring and Assessment.

[CR15] Eira C, Torres J, Miquel J, Vaqueiro J, Soares A, Vingada J (2008). Trace element concentrations in *Proteocephalus macrocephalus* (Cestoda) and *Anguillicola crassus* (Nematoda) in comparison to their fish host, *Anguilla anguilla* in Ria de Aveiro. Portugal. Science of the Total Environment.

[CR16] Eissa I, Gehan I, Wafeek M, Nashwa A (2012). Bioremediation for heavy metals in some Red Sea fishes in Suez, Egypt. SCVMJ.

[CR17] Ezzat S, ElKorashey R, Sherif M (2012). The economical value of nile tilapia fish *Oreochromis niloticus* in relation to water quality of Lake Nasser. Egypt. Journal of American Science.

[CR18] Hassan A, Al-Zanbagi N, Al-Nabati E (2016). Impact of nematode helminthes on metal concentrations in the muscles of Koshar fish, *Epinephelus summana*, in Jeddah, Saudi Arabia. The Journal of Basic & Applied Zoology.

[CR19] Hassan A, Moharram S, El Helaly H (2018). Role of parasitic helminths in bioremediating some heavy metal accumulation in the tissues of *Lethrinus mahsena*. Turkish Journal of Fisheries and Aquatic Sciences.

[CR20] Hassan AA, Akinsanya B, Olakolu FC (2013). Heavy metal concentrations in nematode parasites of *Clarias gariepinus* and *Synodontis clarias* from Lekki Lagoon, Lagos, Nigeria. Journal of Marine Science: Research and Development.

[CR21] Hassanine RE-S, Al-Hasawi Z, Hariri M, Touliabah HE-S (2017). *Sclerocollum saudii* Al-Jahdali, 2010 (Acanthocephala: Cavisomidae) as a sentinel for heavy-metal pollution in the Red Sea. Journal of Helminthology.

[CR22] Heath AG (2018). Water pollution and fish physiology.

[CR23] Hossain MB, Tanjin F, Rahman MS, Yu J, Akhter S, Noman MA, Sun J (2022). Metals bioaccumulation in 15 commonly consumed fishes from the lower Meghna river and adjacent areas of Bangladesh and associated human health hazards. Toxics.

[CR24] Ibrahim, A. T. A., Wassif, E. T., & Alfons, M. S. (2016). *Cyto and genotoxicity biomarkers in erythrocytes of Oreochromis niloticus under the impact of sewage water.* The 8th International Conference for Development and the Environment in the Arab world, March, 22–24.

[CR25] Kapustka, L. A. (2004). *Issue paper on the ecological effects of metals*. US Environmental Protection Agency, Washington, DC, p 77

[CR26] Keke UN, Mgbemena AS, Arimoro FO, Omalu IC (2020). Biomonitoring of effects and accumulations of heavy metals insults using some helminth parasites of fish as bio-indicators in an Afrotropical stream. Frontiers in Environmental Science.

[CR27] Kelle, H., Ngbede, E., & Uju, O. V. (2018). Determination of heavy metals in fish (*Clarias gariepinus*) organs from Asaba Major Markets, Delta State, Nigeria. *Journal of Chemical Society of Nigeria, 43*(1).

[CR28] Kenšová R, Čelechovská O, Doubravová J, Svobodová Z (2010). Concentrations of metals in tissues of fish from the Věstonice reservoir. Acta Veterinaria Brno.

[CR29] Khaleghzadeh-Ahangar, H., Malek, M., & McKenzie, K. (2011). The parasitic nematodes *Hysterothylacium* sp. type MB larvae as bioindicators of lead and cadmium: A comparative study of parasite and host tissues. *Parasitology, 138*(11), 1400–1405. 10.1017/S0031182011000977 21816122

[CR30] Khan R, Thulin J (1991). Influence of pollution on parasites of aquatic animals. Advances in Parasitology.

[CR31] Leite LA, Pedro NH, de Azevedo RK, Kinoshita A, Gennari RF, Watanabe S, Abdallah VD (2016). *Contracaecum* sp. parasitizing *Acestrorhynchus lacustris* as a bioindicator for metal pollution in the Batalha River, southeast Brazil. Science of the Total Environment.

[CR32] Malek M, Haseli M, Mobedi I, Ganjali M, MacKenzie K (2007). Parasites as heavy metal bioindicators in the shark *Carcharhinus dussumieri* from the Persian Gulf. Parasitology.

[CR33] Marcogliese, D. J., Gendron, A. D., Plante, C., Fournier, M., & Cyr, D. (2006). Parasites of spottail shiners (*Notropis hudsonius*) in the St. Lawrence River: Effects of municipal effluents and habitat. *Canadian Journal of Zoology, 84*(10), 1461–1481.

[CR34] Mazhar R, Shazili NA, Harrison FS (2014). Comparative study of the metal accumulation in *Hysterothalycium reliquens* (nematode) and *Paraphilometroides nemipteri* (nematode) as compared with their doubly infected host, *Nemipterus peronii* (Notched threadfin bream). Parasitology Research.

[CR35] Mehana E-SE, Khafaga AF, Elblehi SS, Abd El-Hack ME, Naiel MA, Bin-Jumah M, Othman SI, Allam AA (2020). Biomonitoring of heavy metal pollution using acanthocephalans parasite in ecosystem: An updated overview. Animals.

[CR36] Morsy K, Bashtar A-R, Abdel-Ghaffar F, Mehlhorn H, Al Quraishy S, El-Mahdi M, Al-Ghamdi A, Mostafa N (2012). First record of anisakid juveniles (Nematoda) in the European seabass *Dicentrarchus labrax* (family: Moronidae), and their role as bio-indicators of heavy metal pollution. Parasitology Research.

[CR37] Nachev, M., Schertzinger, G., & Sures, B. (2013). Comparison of the metal accumulation capacity between the acanthocephalan *Pomphorhynchus laevis* and larval nematodes of the genus *Eustrongylides* sp. infecting barbel (*Barbus barbus*). *Parasites & Vectors, 6*(1), 1–8. 10.1186/1756-3305-6-21 PMC3558458 23332036

[CR38] Nachev M, Sures B (2015). Environmental parasitology: Parasites as accumulation bioindicators in the marine environment. Journal of Sea Research.

[CR39] Najm M, Fakhar M (2015). Helminthic parasites as heavy metal bioindicators in aquatic ecosystems. Medical Laboratory Journal.

[CR40] Otachi EO, Körner W, Avenant-Oldewage A, Fellner-Frank C, Jirsa F (2014). Trace elements in sediments, blue spotted tilapia *Oreochromis leucostictus* (Trewavas, 1933) and its parasite *Contracaecum multipapillatum* from Lake Naivasha, Kenya, including a comprehensive health risk analysis. Environmental Science and Pollution Research.

[CR41] Otitoloju, A. A. (2002). Evaluation of the joint-action toxicity of binary mixtures of heavy metals against the mangrove periwinkle *Tympanotonus fuscatus* var *radula* (L.). *Ecotoxicology and Environmental safety, 53*(3), 404–415. 10.1016/s0147-6513(02)00032-5 12485585

[CR42] Palíková M, Baruš V (2003). Mercury content in *Anguillicola crassus* (Nematoda) and its host *Anguilla anguilla*. Acta Veterinaria Brno.

[CR43] Pascual S, Abollo E (2003). Accumulation of heavy metals in the whaleworm *Anisakis simplex* sl (Nematoda: Anisakidae). Journal of the Marine Biological Association of the United Kingdom.

[CR44] Radwan M, Abbas M, Afifi M, Mohammadein A, Al Malki J (2022). Fish parasites and heavy metals relationship in wild and cultivated fish as potential health risk assessment in Egypt. Frontiers in Environmental Science.

[CR45] Rashed M (2001). Monitoring of environmental heavy metals in fish from Nasser Lake. Environment International.

[CR46] Rauf, A., & Javed, M. (2007). Copper-toxicity to water and plankton in the river Ravi, Pakistan. *International Journal of Agriculture and Biology (Pakistan)*.

[CR47] Ravera O, Beone GM, Trincherini PR, Riccardi N (2007). Seasonal variations in metal content of two *Unio pictorum mancus* (Mollusca, Unionidae) populations from two lakes of different trophic state. Journal of Limnology.

[CR48] Roesijadi, G. (1994). Metal regulation in aquatic animals: Mechanism of uptake, accumulation and release. *Molecular mechanisms in aquatic toxicology.*, 387–420.

[CR49] Samuel D, George P (2000). Mineral constituents of freshwater fish *Channa striatus* infected with acanthocephalan parasite *Pallisentis nagpurensis*. Indian Journal of Fisheries.

[CR50] Shahat MA, Amer OSO, AbdAllah AT, Abdelsater N, Moustafa MA (2011). The distribution of certain heavy metals between intestinal parasites and their fish hosts in the River Nile at Assuit Province. Egypt. the Egyptian Journal of Hospital Medicine.

[CR51] Sures B (2003). Accumulation of heavy metals by intestinal helminths in fish: An overview and perspective. Parasitology.

[CR52] Sures B (2004). Environmental parasitology: Relevancy of parasites in monitoring environmental pollution. Trends in Parasitology.

[CR53] Sures B, Nachev M, Selbach C, Marcogliese DJ (2017). Parasite responses to pollution: What we know and where we go in ‘Environmental Parasitology’. Parasites & Vectors.

[CR54] Sures B, Siddall R (1999). *Pomphorhynchus laevis*: The intestinal acanthocephalan as a lead sink for its fish host, chub (*Leuciscus cephalus*). Experimental Parasitology.

[CR55] Sures, B., & Siddall, R. (2001). Comparison between lead accumulation of *Pomphorhynchus laevis* (Palaeacanthocephala) in the intestine of chub (*Leuciscus cephalus*) and in the body cavity of goldfish (*Carassius auratus auratus*). *International Journal for Parasitology, 31*(7), 669–673. 10.1016/S0020-7519(01)00173-4 10.1016/s0020-7519(01)00173-4 11336747

[CR56] Sures B, Siddall R, Taraschewski H (1999). Parasites as accumulation indicators of heavy metal pollution. Parasitology Today.

[CR57] Sures B, Taraschewski H, Jackwerth E (1994). Lead content of *Paratenuisentis ambiguus* (Acanthocephala), *Anguillicola crassus* (Nematodes) and their host *Anguilla anguilla*. Diseases of Aquatic Organisms.

[CR58] Szefer P, Rokicki J, Frelek K, Skóra K, Malinga M (1998). Bioaccumulation of selected trace elements in lung nematodes, *Pseudalius inflexus*, of harbor porpoise (*Phocoena phocoena*) in a Polish zone of the Baltic Sea. Science of the Total Environment.

[CR59] Tellez M, Merchant M (2015). Biomonitoring heavy metal pollution using an aquatic apex predator, the American alligator, and its parasites. PLoS ONE.

[CR60] Thabit, H., & Abdallah, E. S. H. (2022). Morphological and molecular identification of third-stage *Contracaecum* larvae (Nematoda: Anisakidae) parasitizing Nile perch *Lates niloticus* in Egypt. *Aquaculture Research*.

[CR61] Uçkun AA (2017). Ecotoxicological evaluation of pesticide pollution in Ataturk dam Lake (Euphrates River), Turkey. Turkish Journal of Fisheries and Aquatic Sciences.

[CR62] Vidal-Martínez V, Wunderlich A (2017). Parasites as bioindicators of environmental degradation in Latin America: A meta-analysis. Journal of Helminthology.

[CR63] Wakawa R, Uzairu A, Kagbu J, Balarabe M (2008). Impact assessment of effluent discharge on physico-chemical parameters and some heavy metal concentrations in surface water of River Challawa Kano, Nigeria. African Journal of Pure and Applied Chemistry.

[CR64] Zimmermann S, Menzel CM, Berner Z, Eckhardt J-D, Stüben D, Alt F, Messerschmidt J, Taraschewski H, Sures B (2001). Trace analysis of platinum in biological samples: A comparison between sector field ICP-MS and adsorptive cathodic stripping voltammetry following different digestion procedures. Analytica Chimica Acta.

