# Correction to: Biomonitoring of heavy metals using *Contracaecum quadripapillatum* (Nematoda) in comparison to its fish host, *Lates niloticus*, from the Nile River, Egypt

**DOI:** 10.1007/s10661-023-11415-2

**Published:** 2023-06-03

**Authors:** Hasnaa Thabit

**Affiliations:** Department of Zoology and Entomology, Faculty of Science, Assiut University, Assiut, PO 71526 Egypt

**Correction to: Environmental Monitoring and Assessment (2023) 195:530**

**https://doi.org/10.1007/s10661-023-11156-2**

Page 8 of 13: Results, the format of Table 4 is incorrect.

The corrected Table 4 is shown below.

**Table 4:** Pearson correlation coefficients (*r*) between parasite number of *Contracaecum quadripapillatum* and metal concentrations in tissues of parasitic larva and its host, *Lates niloticus*

Parasite Number	Cd	Cu	Fe	Mn	Ni	Pb	Zn
Parasite tissue							
*r*	**−0.558**	0.378	**0.727**	0.231	**0.629**	**0.743**	0.343
*P* value	**0.031**	0.165	**0.002**	0.406	**0.012**	**0.002**	0.210
Liver							
*r*	0.392	0.043	−0.344	−0.233	−0.420	0.124	−0.093
*P* value	0.149	0.878	0.210	0.403	0.119	0.659	0.742
Kidney							
*r*	**0.537**	0.500	**0.774**	**0.977**	**0.759**	0.308	**0.818**
*P* value	**0.039**	0.058	**0.001**	**<0.0001**	**0.001**	0.264	**<0.0001**
Muscles							
* r*	0.480	−0.034	0.297	−0.299	−0.129	**−0.626**	**−0.882**
*P* value	0.070	0.905	0.282	0.279	0.647	**0.013**	**<0.0001**

Bold cells indicate significant values of correlation (*r*)

- Page 5 of 13: Results, the second paragraph.

- Contracaecum larvae had metal concentrations in the following order: Fe > Zn > Ni > Mn > Pb > Cu > Cd, whereas the liver had the following order: Fe > Zn > Ni > Cu > Mn > Pb > Cd; in both kidney and muscles was Fe > Zn > Ni > Mn > Cu > Pb > Cd (Fig. 2). It is incorrect.

The greater than signs are not superscript and should be read and corrected as the below.

*Contracaecum* larvae had metal concentrations in the following order: Fe > Zn > Ni > Mn > Pb > Cu > Cd, whereas the liver had the following order: Fe > Zn > Ni > Cu > Mn > Pb > Cd; in both kidney and muscles was Fe > Zn > Ni > Mn > Cu > Pb > Cd (Fig. 2).

- Page 5 of 13: Results, the third paragraph

The BAFs showed the ability of the parasite to accumulate metals relative to other host tissues under study as shown in Table 2. Except for Cu and Fe, all metals had higher mean BAFs in Contracaecum larvae (>1) than in the host liver. It is incorrect.

The greater than sign is not superscript and should be read and corrected as the below.

The BAFs showed the ability of the parasite to accumulate metals relative to other host tissues under study as shown in Table 2. Except for Cu and Fe, all metals had higher mean BAFs in *Contracaecum* larvae (>1) than in the host liver.

- Page 5 of 13: Results, the 5th paragraph

Pearson correlation coefficients between the parasite number of C. quadripapillatum and metal concentrations in the parasite and fish tissues are shown in Table 4. The number of parasitic larvae was associated positively with Fe, Ni, and Pb concentrations and negatively with the Cd concentration.

It should read and corrected as the below.

Pearson correlation coefficients between the parasite number of *C. quadripapillatum* and metal concentrations in the parasite and fish tissues are shown in Table 4. The number of parasitic larvae was associated positively with Fe, Ni, and Pb concentrations and negatively with the Cd concentration in the parasite tissue.

- Page 1 0f 13: Abstract

The infrapopulation size of C. quadripapillatum correlated with metal concentrations in different host tissues, especially the kidney, while the correlations between metal levels in the tissues of both parasite and fish organs exhibit different patterns in each organ.

It should read and corrected as the below.

The infrapopulation size of *C. quadripapillatum* correlated with metal concentrations in the parasite and different host tissues, especially the kidney, while the correlations between metal levels in the tissues of both parasite and fish organs exhibit different patterns in each organ.

- Page 2 0f 13: Introduction

As a result, higher metal levels in the host muscle and a lower bioaccumulation factor reflect long-term exposure (Sures & Siddall, 2001). It is incorrect.

It should read and corrected as the below.

As a result, higher metal levels in the host muscles and a lower bioaccumulation factor reflect long-term exposure (Sures & Siddall, 2001).

- Page 10 0f 13: Discussion, the 6th paragraph

On the other hand, some metal concentrations in parasite tissue such as Mn and Pb exhibit positive and negative associations respectively with their concentrations in the fish muscle. It is incorrect.

It should read and corrected as the below.

On the other hand, some metal concentrations in parasite tissue such as Mn and Pb exhibit positive and negative associations respectively with their concentrations in the fish muscles.

- Page 10 0f 13: Discussion, the 6th paragraph

In contrast, the present results recorded a significant negative relationship for Pb and Zn in the muscle and a significant positive relationship for Cd, Fe, Mn, Ni, and Zn in the kidney with C. quadripapillatum infrapopulation size. It is incorrect.

It should read and corrected as the below.

In contrast, the present results recorded a significant negative relationship for Pb and Zn in the muscles and a significant positive relationship for Cd, Fe, Mn, Ni, and Zn in the kidney with *C. quadripapillatum* infrapopulation size.

